# The influence of cognitive reserve on ERP measures of selective visual attentional processing in older adults after mild traumatic brain injury

**DOI:** 10.1371/journal.pone.0316673

**Published:** 2025-01-06

**Authors:** Sebastián A. Balart-Sánchez, Mayra Bittencourt, Seyedehzahra Jalili, Joukje van der Naalt, Natasha M. Maurits

**Affiliations:** Department of Neurology, University Medical Center Groningen, University of Groningen, Groningen, The Netherlands; Mae Fah Luang University School of Anti Aging and Regenerative Medicine, THAILAND

## Abstract

**Objective:**

Older adults have an increased risk of developing persistent cognitive complaints after mild traumatic brain injury (mTBI). Yet, studies exploring which factors protect older adults with mTBI from developing such complaints are rare. It has been suggested that one such factor may be cognitive reserve (CR), but it is unknown how CR influences cognition in this patient category. Here, we therefore study how CR influences brain processes during a task that taps into attention, an important cognitive function.

**Methods:**

We studied 17 older adults (13 males, mean 68.18 (SD 5.08) years old) at the subacute stage after mTBI and 19 age- and CR-matched participants without mTBI; 9 males, mean 67.79 (SD 5.36) years old) performing a selective visual attentional processing task while recording EEG. The P2 brain event-related potential component was obtained by averaging over electrodes in the fronto-central region of interest and its amplitude and latency were derived as neural correlates of attentional processing. The inverse efficiency score (IES) was derived from accuracy and reaction times as a measure of performance. To investigate the effect of CR on performance and P2 component characteristics, three separate mixed model repeated measures analyses of covariance (RM-ANCOVA) were performed.

**Results:**

Performance did not significantly differ across groups or task conditions, nor was it significantly influenced by CR. Main effects of CR illustrated that the P2 latency was delayed (p = .03) and the P2 amplitude increased (p = .02) with higher CR across groups. Furthermore, CR correlated positively with P2 latency in both groups (older adults without mTBI: r = .370, p = .005, older adults with mTBI: r = .287, p = 0.041), and with P2 amplitude in the older adults with mTBI (r = .595-.636, p<0.001-.011). We found no main or interaction effects of group or task condition on P2 characteristics.

**Conclusion:**

Older adults with mTBI with higher CR employ more brain resources than older adults with mTBI with lower CR, accompanied by slower processing, suggesting that it may have resulted in similar performance at a selective visual attentional processing task. To better interpret these findings in the context of persistent complaints and establish that higher CR in these patients may result in better performance, our study needs to be repeated with more participants.

## Introduction

The yearly incidence of traumatic brain injury (TBI) cases worldwide is estimated to be 69 million, from which 81% concerns mild traumatic brain injury (mTBI), making mTBI the most common type of TBI [1, 2].

The majority of mTBI patients (80%) will recover within a month [3], while a subgroup of mTBI patients will have persistent post-injury complaints for weeks that can evolve into a post-concussive syndrome (PCS) that can last for years [4]. Age seems to be a crucial factor in developing PCS, as mTBI during childhood and adolescence [5] or older age [6] is linked to an increased risk of developing PCS. A possible explanation is that older adults are more frail than younger groups due to lower physical capacity and multiple pre-existing comorbidities due to ageing [7, 8], leading to worse outcomes and higher mortality [9]. Although older adults account for half of the emergency visits related to TBI [10], the factors protecting them from developing PCS are still not well understood [11]. PCS includes cognitive, emotional, somatic and behavioural symptoms and can therefore greatly impact the quality of life of mTBI patients [12, 13], the more so in older adults with mTBI as older age is a predictor for persistent PCS [14].

Persistent symptoms following mTBI include fatigue and impairments within different domains of cognition, namely deficits in attention, working memory, and information processing speed [15, 16]. Early detection of cognitive impairment after mTBI could allow prompt cognitive behavioral intervention, which has been proven to diminish and prevent the development of worse outcome, including PCS [17]. Therefore, increased cognitive reserve (CR), i.e. better “adaptability of cognitive processes that helps to explain differential susceptibility of cognitive abilities or day-to-day function to brain aging, pathology or brain insult” [18], could theoretically be considered a protective factor that enables to maintain pre-mTBI cognitive functioning [19]. CR has been demonstrated to mediate the clinical neurocognitive manifestation of Alzheimer’s disease (AD) [20], Parkinson’s disease (PD) [21], stroke [22], and TBI [23]. The latter study considered adults with mild to severe TBI, but not specifically older adults. Recent cohort studies suggest that CR mediates the appearance of persistent post-mTBI symptoms from young to older adults [24, 25]. However, in these studies the oldest participants were 65 and 59 years, respectively. Older adults after mTBI are more vulnerable to PCS and tend to have pre-existing conditions that make it harder to predict their outcome [26].

Neuroimaging techniques offer the possibility to study mechanisms underlying these observed positive effects of CR after TBI at brain level. Most studies of the neurobiological basis of CR employ functional magnetic resonance imaging (fMRI), which allows good spatial localization but lacks temporal resolution [27]. Electroencephalography (EEG), on the other hand, allows researchers to study the neural correlates of CR with high temporal resolution, albeit at lower spatial resolution [28]. Event-related potentials (ERPs) offer the possibility to assess cognitive function. In particular, the P2 component has been studied as a neural correlate of information processing and attention [29, 30]. Attentional processing is one of the earliest cognitive processes affected by normal brain aging [31], making older adults at higher risk of attentional dysfunction after mTBI [32]. The P2 latency, in particular, is considered a neural correlate of visual attention allocation as it is sensitive to all incoming stimuli, evaluation, and preparation for subsequent action [33]. A study comparing younger and older adults found that P2 peak latencies were increased with age, suggesting age-related delayed attentional control [34]. In addition, P2 amplitude has been shown to be influenced by visual stimulus characteristics, suggesting that an increased P2 amplitude is related to the use of more neural attentional resources being used during perceptual processing [35]. The P2 component has also shown promise to be used as a diagnostic measure of attentional processing for post-brain surgery patients [36], AD patients [37] and more recently for TBI veterans [38].

Here, to get a better understanding of the factors that protect older adults with mTBI from developing cognitive symptoms in the subacute stage, we investigate how CR affects the impact of mTBI in older adults on selective visual attentional processing and hypothesize that higher CR will be protective against performance deterioration after mTBI; i.e., that higher CR will correlate with better performance. To this end, a visual selective attention task was examined in older adults with mTBI, using the P2 ERP component as a neural correlate of such visual attentional processing. We hypothesize that higher CR will correlate with shorter P2 latency as a measure of better processing efficiency and with higher P2 amplitude as a measure of increased neural allocation of resources. Results were compared with older adults without mTBI.

## Methods

### Participants

Older adults with mTBI were included at the emergency department of the University Medical Center Groningen (UMCG), a level one trauma center in the Netherlands, between January 2019 and October 2020 as part of an ongoing cohort study on outcome after mTBI in older adults (‘Recovery and CONNECTivity in older adults after cerebral trauma’ or ReCONNECT Study). The older control participants without mTBI (non-mTBI group) were age, CR-, and sex-matched on group level to the mTBI patients, meaning that groups did not differ significantly on these characteristics. Non-mTBI older adults were recruited via local newspaper advertisements and notice boards in the UMCG. All included participants had to be 60 years or older. The age of 60 was chosen in accordance with the World Health Organization’s ‘World Report on Ageing and Health’ in which people aged 60 years or older are regarded as older adults [39]. This age limit is also in line with a recent review on the long term consequences of mTBI in older adults [40]. The general inclusion criteria for older adults with mTBI were a Glasgow Coma Scale (GSC) score between 13–15 and loss of consciousness (LOC) and/or post-traumatic amnesia (PTA) according to the ACRM diagnostic criteria [41], including patients with abnormal neuroimaging findings as having mTBI. Exclusion criteria for all participants were psychiatric disorders, drug or alcohol addiction, or previous admittance to a hospital due to TBI. Additionally, all participants needed to have unimpaired or corrected to normal vision according to a self-report questionnaire. Furthermore, they had to have the capability to perform a dual hand motor task while sitting behind a computer and understand the Dutch language. Finally, participants had to correctly execute a trial of the experiment before moving forward with the full experiment.

The ReCONNECT study was approved by the local medical ethical committee of the UMCG (protocol number METc 2017.666) and was executed in compliance with the Declaration of Helsinki (2013). All participants provided written informed consent prior to inclusion. All participants had equal access to care at the time of mTBI and during their recovery.

### Experimental task paradigm

The selective attention task that was employed is adapted from a similar task used in previous fMRI experiments from our research group [42, 43]. First, a target letter was shown for 5000 ms, followed by a fixation cross (+) for 5000 ms in the middle of the screen delineating four quadrants. In this screen, two hashtags in either the bottom left and upper right or upper left and bottom right quadrants indicated the relevant ‘diagonal’ for each block. This screen was followed for 300 ms by another screen with the same fixation cross and four quadrants, now displaying four letters, one in each quadrant. The participant was instructed to press the right button of a button box with the index finger of the right hand when the target letter appeared in one of the quadrants of the relevant diagonal (Target, T) and to press the left button of the button box with the index finger of the left hand otherwise, i.e. if the target letter appeared in one of the quadrants of the irrelevant diagonal (Irrelevant-target, IT) or if the target letter wasn’t present (No-Target, NT). The entire run consisted of four blocks, of 63 trials each. The interstimulus interval varied randomly between 2000 and 2500 ms.

The task was built using E-Prime 2.0 software (Psychology Software Tools, Pittsburgh, PA, United States) and presented on a computer screen at 60 cm distance from the participant’s eyes. The triggers of all stimuli and responses were transmitted to the EEG recording software Brain Vision Recorder v.2.0 through a TriggerBox (both: Brain Products GmbH, Gilching, Germany).

### Behavioural analysis

The triggers associated with stimulus presentation and button presses were used to determine reaction time and accuracy. Task accuracy was defined as the percentage of correct responses that were given between 200 and 1000 ms after stimulus onset. Only correct responses were further analysed. We determined reaction time (RT) in ms and accuracy (ACC) in percentage of correct responses for further analysis. As there is a trade-off between accuracy and speed [44], we combined RT and ACC in the inverse efficiency score (IES) which calculates the ratio between mean reaction time of correct responses and accuracy [44–46], as a measure of performance.

### EEG acquisition

The EEG signal was recorded using a cap with 64 Ag/AgCl active electrodes (ActiCAP/ActiCHamp; Brain Products GmbH, Gilching, Germany) placed according to the international 10–20 system [47] and referenced to Fz with ground at Fpz using a sampling frequency of 250 Hz. Impedances were lowered to below 10 kΩ.

### EEG analysis

Raw EEG data were preprocessed using EEGLab [48] version 2019.1 (sccn.ucsd.edu/eeglab) and ERPLAB [49] version 7.0 (erpinfo.org/erplab) in MATLAB version 2019b (Mathworks Inc, Massachusetts, USA). Raw EEG was first inspected semi-automatically for artifacts (voltage exceeding ± 200μV). When a channel had more than 10% of artifact time points, it was interpolated, while allowing not more than six interpolated channels (10% of the total number of channels) per recording. Otherwise, the whole recording was excluded from further analysis. EEG data were then re-referenced to the average of the 64 scalp channels and filtered using a 12 dB/oct bandpass Butterworth filter between 0.1–45 Hz and a 50 Hz notch filter. Independent Component Analysis (ICA) was used for semi-automatic ocular artifact correction.

Segmentation of epochs was then performed from 200 ms before to 800 ms after stimulus onset to allow analysis of the stimulus-locked P2 ERP component. To ensure that we only included trials in the ERP in which the attention process was actually invoked, we only used segments with correct responses for each condition. Segments were subsequently baseline corrected and averaged per condition (T, IT, NT).

We assessed the attentional P2 component by pooling the electrodes FC1, FC2, F1, F2, and Fz to make a frontocentral region of interest (ROI) that has previously been used to assess the effect of mTBI on ERPs of response inhibition and error processing [50]. We obtained P2 peak latencies from each participant’s ERP for the frontocentral ROI by applying a semi-automatic peak detection algorithm integrated in ERPLAB to the time interval between 180 and 400 ms [51]. We then determined individual P2 peak amplitudes by averaging the amplitude in a 40 ms symmetric interval around peak latency.

### Additional measures

CR was assessed with the Cognitive Reserve Index questionnaire (CRIq: Dutch version [52]) scoring three domains independently: education, working activity, and leisure time. These results were then combined in a composite CR score, the cognitive reserve index (CRI [53]). In a systematic review of measurement properties of CR questionnaires in adults, the CRIq was considered to have fair evidence of content validity in healthy and pathological populations [54]. The CRIq has also been translated to several other languages such as Chinese [55], Turkish [56], and Arabic [57] and all report good validity and test-reliability for the CRIq. Handedness was assessed with the Annett Handedness Scale [58].

### Statistical analysis

All statistical analyses were performed using SPSS version 26 (IBM Corp., Armonk, NY). Normality of data distributions was tested using the Kolmogorov-Smirnov test. If the data were normally distributed, T-tests were used for group comparisons of demographical data. Otherwise, Mann-Whitney U tests were used. For categorical data Chi-square tests were used.

To investigate the effect of CR on performance and P2 component characteristics, we performed three separate mixed model repeated measures analyses of covariance (RM-ANCOVA) of the performance measure (IES), and amplitude and latency for the P2 component. As the older adults with mTBI were CR-matched, we could also include CR as a covariate. Furthermore, to assess the assumption of homogeneity of regression slopes for each dependent variable, we first built three RM-ANCOVA models that included the interaction between the group variable and the CR covariate. If the group×CR interaction was not significant, implying similar regression slopes, we continued to the full-factorial RM-ANCOVA without the interaction term to investigate our main research question. To verify sphericity of the dependent variables in the RM-ANCOVA, we employed Mauchly’s test. In case sphericity was violated, we applied the Greenhouse-Geisser correction. Similarly, in case Levene’s test indicated a violation of the assumption of homogeneity of error variances, we applied a log(10) transformation as a correction. In all models, the between-subject factor was group (mTBI and HC), while the within-subject factor was task condition (T, IT and NT). CRI was included in all models as a covariate. To control for multiple comparisons we used Bonferroni correction, and as a measure of effect size, we calculated η^2^. Here, we considered the following threshold values: 0.01, 0.06 and 0.14 for small, medium and large effects, respectively [59]. Finally, Pearson correlation analysis was employed to investigate the relationship between CR (CRI) and performance (IES), as well as P2 component characteristics, for each group and task condition. Here, we considered 0.25 < r < 0.5 as weak correlation, 0.5 < r < 0.75 as moderate correlation and r > 0.75 as strong correlation. A significance level of alpha = 0.05 was assumed for all statistical tests.

## Results

### Demographics

We initially included 22 older adults with mTBI, of which five participants were subsequently excluded due to excessive EEG artifacts. No older adults without mTBI were excluded for this reason. Hence, we included 17 older adults with mTBI, as well as 19 age-, sex-, and CR-matched older adults without mTBI for further analysis. Demographic characteristics and CR estimates are provided in Table 1. Groups did not differ significantly for age, handedness, sex, or CR. The mTBI group consisted of more males (76.47%) than females, which is in line with the typical sex distribution of mTBI patients [60].

**Table 1:** Demographic characteristics for older adults with and without mild traumatic brain injury.

	Older adults with mTBI	Older adults without mTBI	*p*-value
	n = 17	n = 19	
Sex (m/f)	13/4	9/10	.074^a^
Handedness (R/L)	15/2	19/0	.124^a^
Age (years)	68.18 (5.08)	67.79 (5.36)	.826^b^
CRI	120.71 (22.44)	132.84 (14.06)	.057^b^
CRI-Education	117.59 (16.37)	125.53 (17.89)	.176^b^
CRI-Working Activity	108.94 (18.62)	118.73 (18.73)	.126^b^
CRI-Leisure Time	120.53 (30.15)	129.98 (12.63)	.245^b^
GCS-score ED (13/14/15)	2/4/11	N/A	N/A
Loss of consciousness			
(Unknown/No/<5 min/<30 min)	7/2/5/3	N/A	N/A
Post-traumatic amnesia			
(No/<1 hr/<24 hrs)	4/5/8	N/A	N/A
Head CT abnormalities (Y/N)	9/8	N/A	N/A
Place of trauma			
(Traffic/Home/Work)	9/3/5	N/A	N/A
Days from injury to EEG	37.06 (9.04)	N/A	N/A

Values are provided as mean (standard deviation). mTBI: mild traumatic brain injury, m: male, f: female, R: right, L: left, Y: yes, N: no, N/A: not applicable. CRI: Cognitive Reserve Index, GCS: Glasgow Coma Scale, ED: Emergency Department, EEG: Electroencephalography.

^a^: Chi-square,

^b^: *t*-test,

*: p < .05.

### Homogeneity of error variances and regression slopes

There was a violation of homogeneity of error variances for the P2 amplitude for the target (T) condition, which could be corrected succesfully with a logarithmic transformation. For easier comparison of P2 amplitude across conditions, this transformation was applied to the NT and IT conditions, as well. The assumption of homogeneity of regression slopes was not violated for any of the dependent variables, as the Group×CR interaction effect was always not significant (see Table 2).

**Table 2:** Statistical results for testing homogeneity of regression slopes.

Outcome variable	Interaction	F	Significance
IES	Group×CR	0.022	.884
P2 Latency		0.666	.42
Log_10_(P2 Amplitude)		2.705	.11

CR: cognitive reserve, IES: inverse efficiency score, F: F-score, result of the omnibus test for between-subject effects.

### Selective attention task performance

Results for task performance are provided in Table 3. There was no significant main or interaction effect of group or CR on IES. However, there was a trend main effect of task condition on IES (F(1.68, 55.327) = 3.18, p = .058, η^2^ = .088 (medium effect size)). Bonferroni-corrected pairwise comparisons showed that performance was worse in the IT condition than in both the T condition (p < .001) and the NT condition (p < .001). This indicates that the task worked as expected; i.e., the IT condition was the most difficult. When running the model without CR as a covariate, there was a main effect of group on IES (p< .001), indicating that group did predict IES before inclusion of CR as a covariate in the model.

**Table 3:** Performance characteristics for older adults with and without mild traumatic brain injury.

	Older adults with mTBI	Older adults without mTBI
	(n = 17)	(n = 19)
Accuracy (%)		
Target	84.9 (9.7)	85.7 (14.7)
Irrelevant Target	80.1 (17.9)	83.8 (16.3)
No Target	90.3 (10.7)	92.3 (14.2)
Reaction Time (ms)		
Target	792.3 (77.2)	763.1 (70.3)
Irrelevant Target	888.5 (96.9)	843.8 (78.0)
No Target	840.8 (106.3)	791.3 (98.4)
IES		
Target	9.5 (1.9)	9.3 (2.4)
Irrelevant Target	12.0 (4.3)	10.7 (3.4)
No Target	9.6 (2.4)	9.0 (3.0)

Values are provided as mean (standard deviation). mTBI: mild traumatic brain injury, IES: Inverse Efficiency Score.

### Attentional P2 ERP component

**Fig 1:**
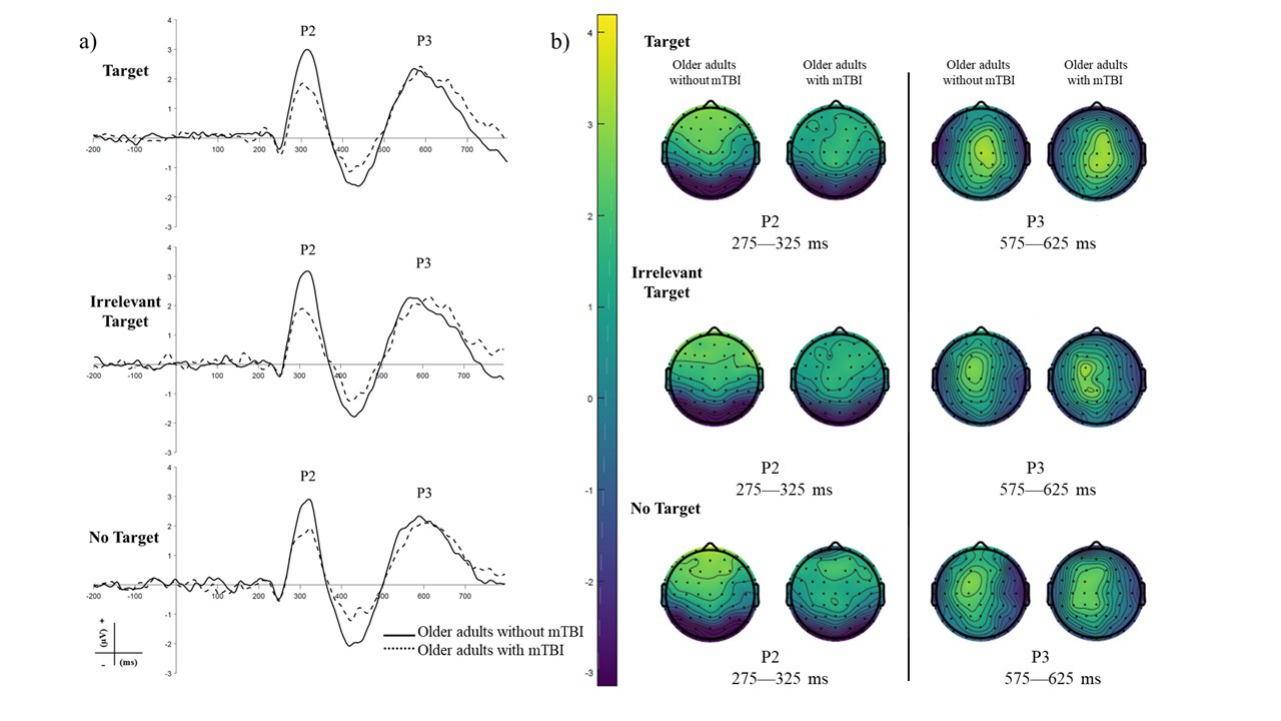
A: Grand average ERP illustrating the attentional P2 at the frontocentral region of interest for the three task conditions for the older adults without mTBI (solid line) and the older adults with mTBI (dotted line). The P3 component is visible at a later latency. Top: target condition, middle: irrelevant target condition and bottom: no target condition. B: Topographical scalp voltage distributions for the P2 and P3 components averaged over 275–325 ms (P2) and 550–650 ms (P2). Left: older adults without mTBI, right: older adults with mTBI.

The grand average ERP at the frontocentral ROI for the three task conditions and each group is illustrated in Fig 1A. After artifact trial removal, 1189 trials remained for the Target condition (per participant median 70, range: 57–79 trials), 1133 trails for the No target condition (per participant median 71, range: 42–83 trials), and 1222 trials for the Irrelevant Target condition (per participant median 78, range: 49–83 trials) for the older adults with mTBI. For the older adults without mTBI the remaining number of trials was 1346 (per participant median 78, range: 39–83 trials), 1330 (per participant median 78, range: 41–83 trials), and 1414 (per participant median 82, range: 44–83 trials), respectively. The P2 component is clearly visible around 300 ms after stimulus onset. Further, the P2 scalp voltage distributions in Fig 1B show that the P2 has the expected fronto-central distribution for each task condition.

**Fig 2:**
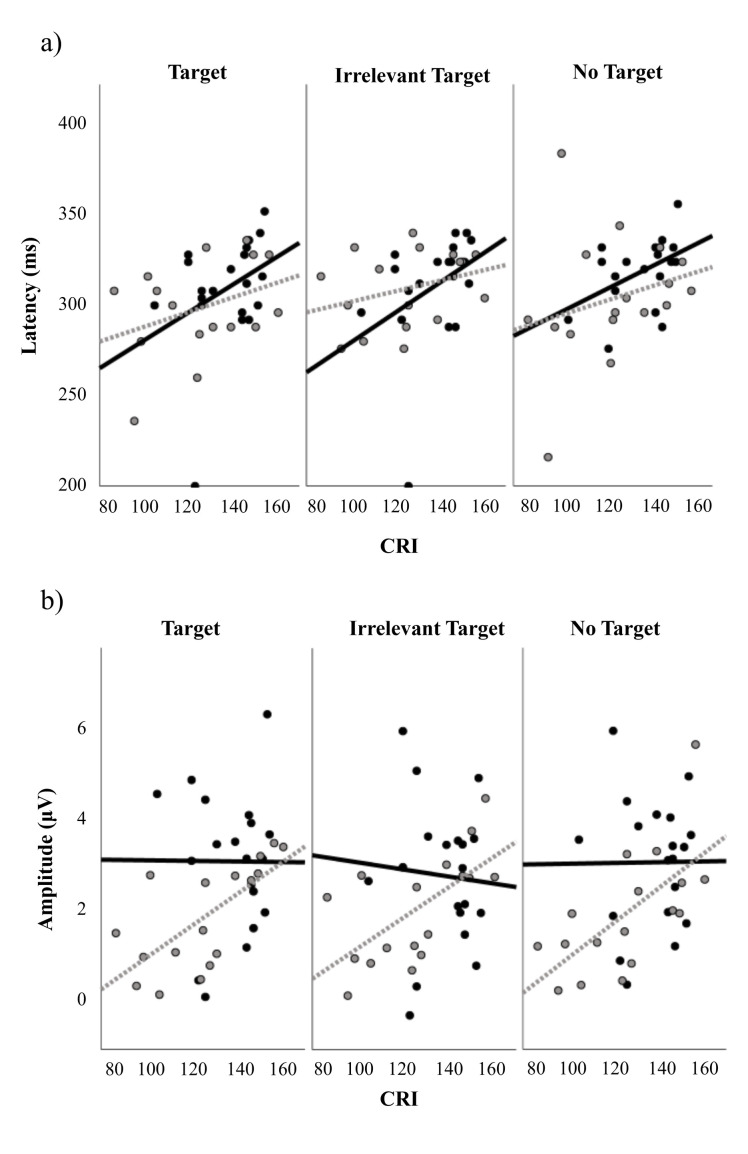
Relation between CRI and a) P2 latency and b) P2 amplitude, for the three task conditions and both groups (black solid line and dots; older adults without mTBI, grey dotted line and dots; older adults with mTBI).

We found a main effect of CR on P2 latency (F(1, 33) = 5.131, p = .03, η^2^ = .135 (medium effect size)), reflecting that P2 latency increased with higher CR (Fig 2a), independent of group or task condition. No other main or interaction effects of task condition or group on P2 latency were found. When running the model without CR as a covariate, there was no main or interaction effect of task condition or group, either.

The P2 amplitude data was logarithmically transformed to correct for inhomogeneity of error variances. We found a main effect of CR on P2 amplitude (F(1, 33) = 6.008, p = .02, η^2^ = .158 (large effect size)), reflecting that P2 amplitude increases with higher CR (Fig 2b). We found no other main or interaction effects of task condition or group on P2 amplitude. When running the model without CR as a covariate, there was no main or interaction effect of task condition or group, either.

#### Correlations

Table 4 provides an overview of the correlations between the CRI and IES and P2 measures. There were no significant correlations between IES and CR estimates. When pooling across task conditions, P2 latency correlated weakly with CR, in both groups (older adults without mTBI: r = .370, p = .005, older adults with mTBI: r = .287, p = 0.041). Such correlation was, however, not found for individual task conditions. P2 amplitude correlated moderately with CR for all individual task conditions, as well as for the pooled task conditions, but only for the older adults with mTBI (r = .595-.636, p<0.001-.011).

**Table 4:** Upper value: Pearson correlations for performance and P2 component measures with the CRI.

	Older adults without mTBI				Older adults with mTBI			
	Target	Irrelevant Target	No Target	Total	Target	Irrelevant Target	No Target	Total
IES	-.077	-.163	.012	-.076	-.097	-.216	-.224	-.169
	.755	.505	.961	.573	.711	.404	.387	.237
P2 latency	.347	.453	.373	.370**	.352	.249	.310	.287*
	.146	.051	.116	.005	.166	.334	.226	.041
Log_10_ P2 amplitude	.147	.131	-.102	.069	.608**	.636**	.595*	.611**
	.549	.594	.688	.614	.009	.006	.011	<.001

Lower value: p-value. CRI: cognitive reserve index, IES: inverse efficiency score, mTBI: mild traumatic brain injury.

*: p < .05.

**: p < .01.

## Discussion

We explored whether CR could protect against the neural impact of mTBI on visual attentional processing in older adults. Specifically, we hypothesized that this protection would manifest as maintained task performance, increased P2 amplitude and shorter P2 latency during the execution of a selective attention task. We found that although there were no significant differences at the behavioural level, increased CR was associated with increased P2 latency and amplitude, relevant to visual attentional processing. When CR was controlled for, there was no significant effect of group on P2 measures or task performance.

We did not find that older adults with mTBI were less efficient than older adults without mTBI for any of the task conditions. That is, they made similar mistakes and needed similar time to react. Our results on task performance for the different task conditions replicate those in a previous study from our group using this task comparing healthy younger and older adults [40]. We found that mTBI did not have a significant effect on P2 component measures when the effect of CR was controlled for. However, some earlier studies did report effects of mTBI, albeit on other cognitive ERP components [61–63]. Discussing these studies is relevant, as the effects of mTBI on ERP component latencies–becoming longer, indicating slower processing—and on amplitudes–becoming lower, reflecting reduced neural resource allocation—would likely be consistent across different ERP components. These earlier studies differ in several, other aspects from the current study providing potential explanations for our seemingly contrasting results. Candrian et al., (2018) found that the NoGo-P3 component amplitude was lower in the acute phase (one week post-injury) but that it normalized between one week and three months post-injury, the latter being more in line with our results that were obtained at 4–6 weeks post-injury. The study of Lachapelle and colleagues (2008) was executed in younger (17–57 years) symptomatic mTBI patients at mean 10.4 (SD 9.0) months post-injury. They found that the P3 component related to visual processing was only affected by mTBI for more complex paradigms and that simpler visual stimuli did not result in a different P3 between mTBI patients and healthy controls. Finally, Shen et al., (2020) found smaller N2 amplitudes in a stop-signal task in younger (20.7–47.1 years) mTBI patients at median 7.8 (range 3.1–30.9) months post-injury who reported a high mean number of 11.4 (SD 10.7) complaints on the Rivermead Post-Concussion Syndrome Questionnaire. As we did not assess symptomatology at 4–6 weeks, it is likely that our sample also included non-symptomatic patients, which may also explain absence of differences, compared to the results of these latter two studies. Furthermore, it is hard to judge how the fact that our attentional paradigm probably tapped into different cognitive processes than the other studies above influences results.

We did find a main effect of CR on P2 amplitude, driven by a significant correlation between CR and P2 amplitude in older adults with mTBI. This finding suggests that older adults with mTBI with higher CR achieve their performance by employing more neural resources, supposedly underlying the observed higher P2 amplitude, in agreement with our hypothesis. Similar findings regarding higher CR and enhanced P2 amplitudes have been found in older adults with higher CR who exhibit increased P2 amplitudes compared to older adults with lower CR during execution of a Stroop interference task [29]. We did not observe an increase in performance as reflected in the IES with higher CR, contrary to our hypothesis. As (weak) inverse relationships between these measures can be observed in general, the lack of such a relationship between CR and performance may be due to the limited power of our study (also see Limitations section).

Regarding neural efficiency, higher CR was associated with a delayed P2 latency across conditions for both older adults with mTBI and older adults without mTBI, in contrast to our hypothesis. In ERP studies, component latencies are considered a measure of processing speed, defined by the efficiency of information flow in the underlying brain network [64], which generally increase with age [65]. In our population, however, CR was not associated with age, so this cannot explain the increased latencies with higher CR. Furthermore, prolonged P2 latencies in older adults in task paradigms that involve attention and working memory have also been associated with better performance [66]. Yet, in our relatively small population we did not find evidence that higher CR may indeed be associated with better performance, as explained above. Hence, we have no conclusive explanation for the positive association between P2 latency and CR [29].

It has been suggested that promptly targeted early interventions after mTBI significantly reduce the risk of developing PCS [17]. However, identifying at the subacute stage which mTBI patients are at risk for unfavourable outcome remains a challenge as until now prediction models achieve unsatisfactory performance [14, 67]. It has been reported before that lower CR is a risk factor for developing PCS [24, 25], albeit in slightly younger patients than in the current study. Here, we provided evidence on the neural mechanisms underlying this finding: older adults with mTBI and higher CR appeared to recruit more neural resources, likely contributing to the observed higher P2 amplitude. However, this increase did not translate into significantly better performance. If our findings are confirmed and expanded by demonstrating increased performance in patients with higher CR in a larger, better powered study, particularly across a range of cognitive tasks, this could enhance our understanding of how higher CR may act as a protective factor against developing PCS after mTBI.

## Limitations and future directions

It has to be acknowledged that the majority of patients had CT-abnormalities which might be higher compared to earlier series. This finding could be related to the fact that part of the patients were included in the COVID period, in which less severely injured patients might not have been admitted to the hospital [68]. This also accounts for the relatively low number of participants in our study. A larger, better powered study is needed to investigate whether, in the context of a selective attention task, older adults with mTBI with higher CR have improved performance compared to those with lower CR and whether this is achieved by employing more neural resources. To further generalize our findings to other cognitive processes that are not tapped into while performing a selective attention task, other cognitive paradigms need to be explored too.

## Conclusion

Older adults with mTBI and higher CR employ more brain resources than older adults with mTBI with lower CR, accompanied by slower processing, resulting in similar performance at a visual attentional processing task. To better interpret these findings in the context of PCS and establish that higher CR in these patients may result in better performance, our study needs to be repeated with more participants and preferably while exploring multiple cognitive paradigms.
